# Feeding the emergence of advanced heart disease in Soweto: a nutritional survey of black African patients with heart failure

**DOI:** 10.5830/CVJA-2011-021

**Published:** 2012-06

**Authors:** Sandra Pretorius, Karen Sliwa, Verena Ruf, Karen Walker, Simon Stewart

**Affiliations:** Soweto Cardiovascular Research Unit, Department of Cardiology, Chris Hani Baragwanath Hospital, University of the Witwatersrand, Johannesburg, South Africa; Soweto Cardiovascular Research Unit, Department of Cardiology, Chris Hani Baragwanath Hospital, University of the Witwatersrand, Johannesburg, South Africa; Soweto Cardiovascular Research Unit, Department of Cardiology, Chris Hani Baragwanath Hospital, University of the Witwatersrand, Johannesburg, South Africa; Baker IDI Heart and Diabetes Institute and Monash University, Melbourne, Australia; Monash University, Melbourne, Australia

**Keywords:** heart failure, Africa, food preferences, malnutrition, salt

## Abstract

**Aim:**

To describe dietary habits and potential nutritional deficiencies in black African patients diagnosed with heart failure (HF).

**Methods and Results:**

Dietary intake in 50 consecutively consenting HF patients (mean age: 47 ± 18 years, 54% female) attending a major hospital in Soweto, South Africa were surveyed using validated quantitative food frequency questionnaires. Food intakes, translated into nutrient data were compared with recommended values. In women, food choices likely to negatively impact on heart health included added sugar [consumed by 75%: median daily intake (interquartile range) 16 g (10–20)], sweet drinks [54%: 310 ml (85–400)] and salted snacks [61%: 15 g (2–17)]. Corresponding figures for men were added sugar [74%: 15 g (10–15)], sweet drinks [65%: 439 ml (71–670)] and salted snacks [74%: 15 g (4–22)]. The womens’ intake of calcium, vitamin C and vitamin E was only 66, 37 and 40% of the age-specific requirement, respectively. For men, equivalent figures were 66, 87 and 67%. Mean sodium intake was 2 372 g/day for men and 1 972 g/day for women, 470 and 294% respectively, of recommended consumption levels.

**Conclusions:**

The nutritional status of black African patients with HF could be improved by recommending healthier food choices and by reducing the intake of sweet drinks and excess salt.

## Summary

Heart failure (HF) has become a major public health problem in that, unlike other cardiovascular diseases, the number of people discharged from hospital with a diagnosis of HF is increasing.^1^ In developed countries, HF can be observed in 2 to 3% of the population and asymptomatic ventricular dysfunction is evident in about 4% of the population.^2^ This will increase with age, and in the 70- to 80-year age group, HF can be observed in between 10 and 20% of people.^2^,^3^ The epidemic of cardiovascular disease (CVD) has probably stabilised in developed countries, but developing countries are increasingly suffering from the emerging burden of CVD.^4^

As populations in South Africa and sub-Saharan Africa undergo economic development, the disease profile shifts and CVD becomes a growing cause of death and disability.^5^ Previously considered a rarity in Africa and predominantly caused by infectious disease or idiopathic dilated cardiomyopathy,^6^ the syndrome of heart failure (HF) has emerged as a challenging public health problem in sub-Saharan Africa.^7^

The Heart of Soweto (HOS) study^8^ has documented a much higher-than-expected burden of modifiable risk factors^9^ and advanced forms of heart disease^10^ linked to epidemiological transition in one of Africa’s largest urban concentrations of black Africans. Data from the HOS study showed that during the period from 2006 to 2008, of the 5 328 *de novo* cases captured with heart disease, 2 505 (47%) of these cases presented with chronic heart failure.^11^,^12^ Ominously, in addition to the ‘traditional’ causes of HF in Africa, such as idiopathic dilated cardiomyopathy, rheumatic fever, HIV-related cardiomyopathy, peripartum cardiomyopathy and hypertensive heart failure, ‘lifestyle’ factors, including hypertension, obesity, dyslipidaemia and type 2 diabetes mellitis (particularly in women), appear to have expanded the pathways to, and burden of, HF in this community.^8^

Although the natural history of HF in Africa is still different from that of high-income countries, it results in the same high level of preventable morbidity and premature mortality.^13^,^14^ In those countries already in the midst of an epidemic of HF, multidisciplinary management programmes targeting the common factors leading to clinical instability have been successfully developed.^15^-^17^ Certain positive measures have been implemented in low- and middle-income countries for disease prevention, including WHO initiatives.^5^ However, inadequate funding hinders efforts to establish adequate multidisciplinary management programmes targeting the common factors leading to heart disease in South Africa.^5^ Moreover, the role of dieticians has been largely confined to patients with lipid disorders, obesity, diabetes and/renal failure.^18^ If, however, nutritional education and promotion of good nutrition could be better understood and recognised to be inclusive of behavioural change, then it will be viewed as a necessary component within contemporary cardiac rehabilitation and self-management programmes.^18^

One cornerstone of HF management particularly relevant to urban Africans affected by HF is dietary modification. For example, sodium restriction (2–3 g/day) is standard therapy in the management of symptomatic chronic HF, and black individuals are particularly responsive to this strategy.^19^,^20^ However, lack of adherence and poor self-care behaviours persist, with dietary indiscretions contributing to a substantial portion (up to 20%) of hospital readmissions.^21^ Specific dietary interventions play an important role in improving health outcomes.^21^,^22^

Three major studies addressing food choices and dietary patterns in adult black South Africans were identified from the literature. However, given the historical rarity of the syndrome, there are very little data to describe the dietary habits of specifically urban African patients with HF. The Dikgale study^23^ examined food choices, nutrient intake and weight status of black adults. The Transition, Health and Urbanisation study (THUSA)^24^ examined the food choices, health status and the effect of urbanisation on a black population. The Black Risk Factor study (BRISK)^25^ examined the risk factors for developing CVD in urban black Africans. Data from these studies show that rural black adults have a very low consumption of fat and a high consumption of carbohydrates, typical of the traditional rural African diet.^23^,^25^

Urbanisation is associated with markedly increased intake of fat, sugar, meat and beverages.^23^-^25^ Although a decrease in the consumption of maize porridge with urbanisation was found, it is still consumed in high amounts by these black population groups.^23^ As the traditional diet is abandoned in favour of a Western diet, food choices shift away from complex carbohydrates and higher fibre to foods high in fat, bringing an increased risk for chronic diseases of lifestyle.^26^ According to Stewart *et al*., data on the population of Soweto have shown a low prevalence for CVD and the underlying risk factors.^8^ This situation however may be changing, as urbanisation and the nutritional transition in South Africa is accompanied by an increase in the CVD risk factors in black Africans.^27^

The overall study aim was therefore to provide a detailed description of the dietary habits and potential nutritional deficiencies in a subgroup of urban black African patients diagnosed with HF, living in Soweto, South Africa, and managed via the Cardiology Unit of the Chris Hani Baragwanath Hospital. It focused on the impact of varied dietary patterns, the poor socio-economic status of many patients and probable lack of awareness of the contribution of poor nutrition to cardiovascular disease. Ultimately, these data will be used to identify key targets for more culturally sensitive support and to argue for a greater role for dieticians in the management of an increasing number of urban black South Africans affected by HF.^12^

## Methods

As part of the previously described Heart of Soweto study,^8^ detailed demographic and clinical data are captured from all individuals with heart disease presenting to the Chris Hani Baragwanath Hospital, Soweto, via a prospective clinical registry. In 2006, this included 1 960 patients presenting with a primary or secondary diagnosis of HF (an average of 162 patients per month). All were diagnosed by echocardiography and specialist cardiological review. This was a prospectively planned study of 50 consecutively consenting black Africans (28 females, 22 males), referred to the Heart Failure Clinic in 2006/7 with a documented diagnosis of HF.

The study was approved by the Human Research Ethics Committee (Medical), University of the Witwatersrand, Johannesburg, M050550. All participants provided written informed consent. The study fully conformed to the principles outlined in the Declaration of Helsinki.

## Dietary instrument, data and nutrient analyses and recommendations

In addition to the detailed clinical and demographic data collected as part of the Heart of Soweto Clinical Registry, an interviewer-administered quantitative food frequency questionnaire (QFFQ) was collected at a point in time when patients had received either limited, or no instructions for a low-sodium, low-fat therapeutic diet for HF.^28^,^29^ A quantitative food frequency questionnaire is a validated questionnaire to determine food choices and consumption. The previously validated QFFQ used in this study was developed by a researcher at Northwest University. This QFFQ has previously been used to evaluate the food choices of the African population living in the North West Province, South Africa, as part of the THUSA study.^30^,^31^

The quantitative QFFQ has been validated via statistical methods in an African population.^30^ It includes 139 types of food and records how often a given type of food is consumed as: time/s per day, per week, per month. It also records preparation methods. Quantities of food eaten were determined in relation to pictures of standardised portions of the most commonly consumed foods (e.g. maize meal porridge, rice, meat, etc.). The researcher also used standardised cups, teaspoons etc. to estimate portion sizes. The patients were also asked to name foods eaten that were not included in the questionnaire and to point out questions that were unclear or difficult to understand.

The QFFQ was administered through interview by the researcher, SP, who is a registered dietician in the Heart Failure Outpatient Clinic, Chris Hani Baragwanath Hospital, and trained in administering the QFFQ.

Food data were translated into nutrient data using the Medical Research Council (MRC) Food Finder 3, 2007, which is based on South African food composition tables. Total dietary starch was calculated from the total amount of carbohydrates minus the sum of total dietary fibre plus added sugars. To assess the consumption of high-sodium foods, data were aggregated to provide percentages of high-sodium foods consumed both daily and weekly.

Collated dietary patterns and nutritional intake data were compared to the South African Food-Based Dietary Guidelines. Importantly, one guideline advises that unrefined or minimally processed starchy foods, such as maize, wheat, sorghum, oats, rice in the form of porridges, breads, pastas, samp, breakfast cereals and other products should be the main food around which the rest of the meal is planned.^32^ Promotion of carbohydrate-rich foods contributes to optimal nutrient intake, particularly in low-income groups. Largely unrefined carbohydrate-rich foods are excellent sources of dietary fibre and provide several important vitamins and minerals.^33^ It is also recommended that in a healthy, balanced diet, protective against chronic diseases of lifestyle, at least 55% of the total energy (%E) should be provided by a variety of carbohydrate-rich foods, with around 30%E provided by fat and 15%E by protein. To provide at least 55%E in an 8 500 kJ diet, at least 275 g carbohydrate should be consumed daily.^32^

## Demographic and clinical data

At the time the QFFQ was administered, body mass was measured with an electronic digital scale, measuring up to 200 kg in graduations of 0.1 kg (Seca 767), and body height was taken with a telescopic measuring rod (Seca 220) attached to the scale, to the nearest 1 mm. Data on the clinical [including left ventricular ejection fraction, New York Heart Association (NYHA) functional class and concurrent diagnoses] and socio-demographic profile (including age, gender and educational status) were collected prospectively.

## Statistical analyses

Data were analysed using SPSS for Windows version 14.0.1 (SPSS Inc, Chicago, Illinois). Normally and non-normally distributed continuous data are given as the mean (standard deviation: SD) and median (interquartile range: IQR), respectively. Categorical data are presented as counts and percentages. Proportional data were compared via the Chi-squared test while all nutrient data were compared via the Mann Whitney U-test according to gender, and actual versus recommended dietary intake. Significance has been accepted as *p* < 0.05 (two-tailed).

## Results

The demographic and clinical profile of the study cohort is shown in Table 1. Reflective of the overall Heart of Soweto study cohort, there were more women (56%) than men. Women were slightly, but not significantly younger than the men and the entire HF cohort was typically two decades younger than that seen in high-income countries. Hypertension and obesity were highly prevalent in both genders. Concurrent diabetes was also common, particularly in men. The majority of patients had left ventricular systolic dysfunction (LVEF < 45%) and symptoms of exercise intolerance and dyspnoea indicative of NYHA functional class II or III.

**Table 1.** Demographic And Clinical Profile Of The Study Cohort

*Socio-demographic profile*	*Men n = 22 (%)*	*Women n = 28 (%)*
Mean age (years)^1^	51 ± 12	47 ± 18
No education	1 (4.5)	4 (14)
1–5 years’ education	5 (23)	7 (25)
6–10 years’ education	15 (68)	16 (57)
Post-matriculation qualifications	1 (4.5)	1 (3.6)
*Risk profile*		
Body mass index (kg/m^2^)^1^	25.2 ± 4.8	26.5 ± 6.4
Hypertension	14 (65)	18 (65)
Diabetes	2 (10)	2 (7.6)
*Heart failure profile*		
NYHA class II	11 (50)	12 (43)
NYHA class III	4 (19)	9 (32)
NYHA class IV	0 (0)	1 (3.6)
Left ventricular ejection fraction^1^	37.3 ± 9.1%	36.4 ± 13.4%

^1^Data are given as mean ± SD or as number (%).

The daily food consumption of the cohort as measured by the QFFQ according to gender is shown in Table 2. Significantly, more women (79%) than men (65%) reported eating brown or wholemeal bread, 75% of women and 48% of men consumed sweets and chocolates, processed meat were eaten by 89% of women and 78% of men, and packet soup was consumed by 57% of women and 43% of men. Women, but few men also reported eating stock cubes, 18% and 4% respectively, possibly since the women were more aware that they were added during food preparation. Conversely, more men than women reported the consumption of takeaway foods, 48 and 32%, respectively. Salted snacks were eaten by 74% of men and 61% of women, while more men than women reported adding salt to cooked food, 91% and 75% respectively. Despite differences in the proportions of men and women selecting certain foods, the median daily intake of foods eaten was broadly similar for men and women. Specific differences included a higher median intake for men of milk and milk products, and for cakes and biscuits (both *p* < 0.05).

**Table 2.** Daily Food Consumption Of Hf Patients According To The Quantitative Food Frequency Questionnaire

	*Men (n = 22)*		*Women (n = 28)*	
*Foods/food groups*	*Proportion (%)*	*Median daily intake (interquartile range)*	*Proportion (%)*	*Median daily intake (interquartile range)*
Maize meal (g)	91	516 (200–750)	93	424 (140–688)
Mabella (g)^1^	52	78 (25–64)	57	111 (55–136)
Oats (g)	26	88 (55–107)	32	80 (50–100)
Potatoes (g)	78	76 (28–91)	86*	59 (28–89)
White bread (g)	22	88 (50–60)	29	73 (38–98)
Brown/wholegrain bread (g)	65	102 (43–120)	79***	87 (60–113)
Cereals: refined (g)	13	13 (13–15)	14	7 (4–10)
Cereals: wholegrain (g)	22	29 (25–30)	21	17 (9–15)
Mageu (ml)^2^	30	208 (43–321)	39	64 (16–71)
Added sugar (g)	74	15 (10–15)	75	16 (10–20)
Sweets and chocolates (g)	48	19 (7–30)	75***	11 (3–12)
Cakes and biscuits (g)	48	45(15–25)	57	7(5–10)*
Cold drinks (sweetened) (ml)	65	439 (71–670)	54*	310 (85–400)
Meat, chicken, fish, eggs (g)	100	150 (105–190)	100	127 (83–168)
Milk and milk products (ml)	87	262 (129–370)	93*	113 (58-145)*
Legumes (g)	43	18 (10–24)	43	18 (9–28)
Fruit (fresh) (g)	100	174 (150–160)	100	147 (40–160)
Vegetables (fresh) (g)	100	76 (40–103)	100	78 (50–91)
Margarine on bread (g)	83	15 (7–20)	75	16 (10–20)
Salt added to cooked food (g)	91	2 (2–2)	75***	2 (2–2)
Salted snacks (g)	74	15 (4–22)	61**	15 (2–17)
Take-away foods (g)	48	23 (10–15)	32***	16 (10–25)
Sauces and condiments	57	7 (2–10)	64	4 (2–5)
Stock cubes	4	1 (1–1)	18***	1 (1–2)
Packet soup	43	3 (1–5)	57**	2 (1–2)
Processed meat	78	35 (8–54)	89**	26 (8–35)

^1^Unrefined porridge made from sorghum; ^2^Dried and broken corn kernels; ^3^A carbohydrate-rich drink made from fermented mealie (maize) meal and malt. Significant difference between men and women, *p < 0.05, **p < 0.01, ***p < 0.001.

Median daily nutrient intake in this HF population group is shown in Table 3. Although men consumed a significantly greater quantity of protein (*p* < 0.05), protein as a percentage of energy was similar (around 13%E) for both men and women. Both men and women consumed high amounts of carbohydrate (47–52%E). Although added sugar intake was low (< 10%E), fibre intake was moderately low, suggesting that many carbohydrate foods eaten came from refined sources, rather than from wholegrain cereals, as recommended. Both women and men consumed < 30%E from fat. Consumption of saturated fat and trans fat was significantly lower in women than men (*p* < 0.05, *p* = 0.001, respectively). Four men (18%) consumed alcohol, with one reporting consumption equivalent to 27 g per day. Only one woman (3.5%) drank alcohol.

**Table 3.** Energy And Daily Nutrient Intake Of HF Patients Based On A Quantitative Food Frequency Questionnaire

	*Daily intake [median (interquartile range)]*	
Nutrient	Men (n = 22)	Women (n = 28)
Energy (kJ)	9 145 (6 857–12 879)	7 472 (5 568–9 478)
Protein (g)	74.0 (101–62)	58.8 (51–66)*
% plant-derived	42.8	43.5
% total energy	13.8	13.4
Total carbohydrate (g)	272 (223–404)	245 (170–336)
% total energy	47.6	52.5
Starch (g)	17.8 (13–27)	11.0 (8–17)
Dietary fibre (g)	20.6 (14–25)	16.2 (13–23)
Added sugars (g)	40.2 (23–76)	33.1 (19–69)
Total fat (g)	65.7 (5–91)	47.4 (39–81)
% total energy	26.6	23.5
Saturated fat (g)	19.9 (16–29)	15.1 (12–20)*
% total energy	8.1	7.5
Monounsaturated fat (g)	22.9 (17–31)	17.0 (14–26)
% total energy	9.3	8.4
Polyunsaturated fat (g)	15.7 (11–22)	12.7 (9–25)
% total energy	6.3	6.3
Total trans fat (g)	.94 (0.62–1.8)	0.46 (0.28–0.67)**
Cholesterol (mg)	308 (177–403)	214 (160–307)

Significant difference between men and women, *p < 0.05, **p = 0.001.

Table 4 indicates mean daily micronutrient intake for men and women. In men, the mean intake of calcium and magnesium and of vitamins C, D, E and folate was inadequate. Mean intakes of these nutrients were also inadequate in women although mean intakes of vitamin D and magnesium were only marginally low. The mean intake of iron in women was only 50% of the level recommended, while mean intakes of riboflavin, vitamin B_6_, pantothenate and niacin were also moderately low.

**Table 4.** Micronutrient Intake Of HF Patients In Relation To Recommended Dietary Intakes

*Micronutrient deficiency*	*Daily intake men*	*Difference from DRI (%)*	*Daily intake women*	*Difference from DRI (%)*
Vitamin D (mcg)	4.5	–0.5 (90)	4.7	–0.3 (6)
Vitamin C (mg)	78	–12 (87)	47	–28 (37)
Magnesium (mg)	361	–59 (86)	292	–29 (9)
Vitamin E (mcg)	10	–5 (67)	9	–6 (40)
Calcium (mg)	655	–345 (66)	411	–789 (66)
Folate (mcg)	227	–173 (57)	187	–213 (53)
Iron (mg)			9	–9 (50)
Riboflavin (mg)			1.0	–0.1 (9)
Vitamin B_6_ (mg)			1.2	–0.1 (8)
Pantothenate (mg)			4.6	–0.4 (8)
Niacin (mg)			13.4	–0.6 (4)
Potassium (mg)			1938	–0.62 (4)
*Adequate intake*				
Sodium (mg)	2.372	+1 872 (470)	1 972	972 +1 472 (294)
Potassium (mg)	2512	+0.512 (150)		
Vitamin B_12_ (mcg)	6.3	+3.9 (260)	6.1	+3.7 (254)
Pantothenate (mg)	6.5	+1.5 (130)		
Biotin (mcg)	39	+9.0 (130)	34	+4 (13)
Iron (mg)	11	+3 (125)		
Riboflavin (mg)	1.5	+0.2 (115)		
Niacin (mg)	18	+2.0 (113)		
Vitamin B_6_ (mg)	1.4	+0.1 (108)		
Thiamine (mg)	1.3	+0.1 (108)	1.1	0 (0)
Vitamin A (RE) (mcg)	949	+49 (105)	970	+270 (39)

**Fig. 1.**
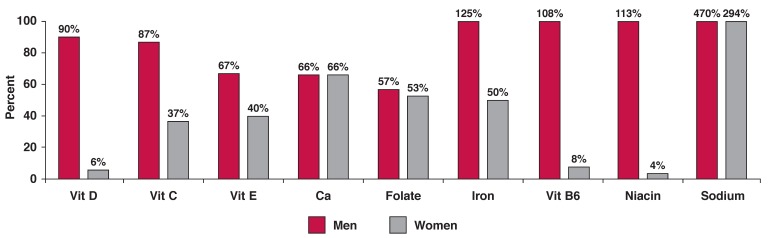
Proportion of men and women consuming more than the recommended daily intake of sodium or with less than the daily recommended intake of selected micronutrients. Significant difference between men and women, *p < 0.01, **p < 0.001.

**Fig. 2.**
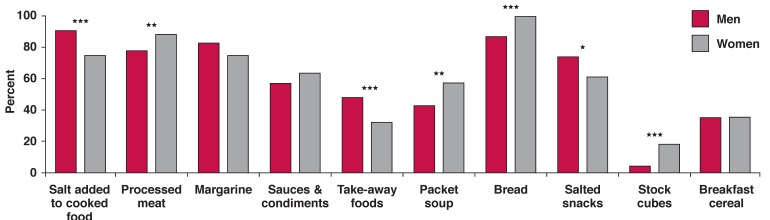
Percentage of men and women consuming the 10 foods contributing most to mean sodium intake in HF patients.

Fig. 1 indicates that often over half of this patient group had inadequate micronutrient consumption, while all the women and the majority of men consumed excessive amounts of sodium. Sodium intake was 470% above recommended intake levels in men and 294% above recommended intake levels in women. As seen in Fig. 2, most sodium came from bread and processed foods. In the body, the ratio of sodium (in extracellular fluid) to potassium (in intracellular fluid) is about 2:3. As seen in Table 4, the intake of potassium in relation to sodium was too low, due to the increased consumption of processed food and the inadequate intake of fruits, vegetables and unrefined cereals.

The likely cost of consuming a healthy diet in Soweto was calculated based on food prices relative to minimum income support available in May 2008. Current food intake required an expenditure of approximately 40% of the current disability grant, which in 2008 was R940 per month. A recommended food intake, where maize meal porridge is supplemented with mabella (coarse), legumes, carrots, spinach, apples, oranges and full-cream milk would require an expenditure of only 30% of this benefit and therefore represents an attractive option both from a financial and health status perspective.

## Discussion

The most significant finding is the inadequate nutrient intake and excessive salt consumption in this high-risk HF patient cohort. Processed and convenience foods contributed to the high intake of salt as well as saturated and trans fatty acids. Low consumption of fruit and vegetables contributed to the low micronutrient and dietary fibre intake. Overall, the pattern of dietary consumption observed is likely to have been a major contributor to the pattern of sub-optimal health outcomes (i.e. premature mortality and recurrent morbid events) found in these patients from Soweto with HF.^8^

High salt intake, particularly in men, was a major problem in this black urban patient group. This, related to a high consumption of bread, processed and take-away foods and the use of high-salt stock cubes and sauces, consistent with North American findings where salt in bread and pre-prepared and cereal foods contributed to around one-quarter of total salt intake.^33^ Possible barriers to adherence to a healthy, low-salt diet in this black population were: lack of knowledge regarding high-salt foods and healthy affordable alternatives, perceptions that meals prepared without added salt were tasteless and boring, and lack of support for dietary change from family members.^34^

Although a salt restriction (2–3 g/day) is standard therapy for HF,^35^ black Sowetans with HF commonly consumed 5–7 g per day.^36^ This indicates the need for higher levels of dietetic education to achieve sodium-restricted diets. At Chris Hani Baragwanath Hospital, 10 registered dieticians currently provide a nutritional service to 2 500 patients; clearly an inadequate ratio of 1:250, instead of the more acceptable ratio of 1:50.^37^

In contrast to the rural areas of South Africa where more ‘traditional’ food patterns still apply, in the urban areas undergoing very rapid epidemiological transition, poor quality ‘Westernised’ diets are common.^26^,^38^ The South African Dietary Guideline (SADG) addresses these nutritional issues, although compliance with recommendations is not readily achieved by disadvantaged urban populations.^38^ The SADG for example recommend servings of ‘meat, fish, chicken or eggs’ should be eaten daily as nutrient-rich sources of high-quality protein. As selection of fatty meats and full-fat dairy foods can increase cardiovascular disease risk, Scholtz and colleagues^39^ suggest a safe daily intake would comprise: 400–500 ml milk, two to three servings of fish and four eggs, and no more than 560 g of meat per week.

In this group of CHF patients, median intake is less than half this amount, presumably as these foods are not affordable. Nevertheless, the proportion of dietary protein was within accepted levels (13%E), although the majority came from plant rather than animal sources, with implications for micronutrient intake. Calcium intake, particularly in women was inadequate. Some more affordable sources of plant protein, notably legumes, rich in many nutrients, were not selected in quantity, suggesting lack of familiarity with preparing meals using these foods.

The total fat intake seen in this HF cohort was within recommended levels (< 30%E) but saturated fat intake was excessive, particularly in men, and was related to choice of poor-quality fatty meat, high-fat dairy foods, cakes and biscuits, and take-away foods. This is consistent with the trend for higher total fat and saturated fat consumption seen with urbanisation throughout South Africa.^40^ This patient group continued to eat more traditional carbohydrate foods such as maize porridge, oats and mabella, but also consumed highly refined carbohydrate foods such as cakes, biscuits, cold drinks, sweets, chocolates and added sugar, liable to increase triglycerides and to promote insulin resistance and obesity.^33^

Fruit and vegetables provide alternative sources of carbohydrates and contain many cardioprotective nutrients,^41^ including potassium (lowers blood pressure), folate (reduces plasma homocysteine), vitamin C and many polyphenolic compounds (with antioxidant activities), and soluble fibre (lowers cholesterol). Green leafy vegetables are also high in magnesium (associated with a lower CVD risk). The SADG therefore recommend an intake of five to eight portions (400–600 g) of fruit and vegetables daily.^41^ Black urban Sowetans with HF however, consumed only around one piece of fruit and one vegetable serving per day. Poor affordability and availability probably accounted for this low intake.^41^,^42^ The 1999 South African National Food Consumption Survey indicated that where household income was less than R12 000 per annum, few foods were found in the house (maize, salt, white sugar, tea, fat/oils, white rice and white bread were most common) and micronutrient intakes were frequently low.^42^

This study has several limitations. Firstly, it was a preliminary investigation, performed in a fairly homogeneous group of HF patients. The study was unable to explore the effects of gender roles (women in Soweto still buy and prepare most of the food), effects of differences in average household income, and seasonal variance or the availability of food. These factors limit the extrapolation of these data to other patient populations. It would be of interest in further studies to explore the effects of the media on exposure to Western processed foods, as well as barriers to knowledge on the selection and preparation of healthier foods. However, data presented here were meticulously collected using validated tools.

## Conclusion

This study found that urbanised black Sowetans with HF have high salt intakes and a nutrient-poor diet, placing them at high risk for deteriorating cardiac function and a premature death. Many poor households remain food insecure, which limits their ability to improve their food choices and their overall management, with potentially life-saving consequences. Nutritional education should therefore focus on foods that are varied, available, affordable, culturally acceptable and popular, as well as consistent with the low-salt, low-fat, high-fibre guidelines.^43^ Home cooking should also be encouraged. By not adding salt to cooking and not eating processed foods high in salt, salt intake can be reduced,^35^,^36^ while dietary compliance can be improved by encouraging use of herbs and spices and by providing recipes for appealing low-salt foods. Patient education on reading food labels and recognising high-salt foods should also be expanded.

Recommendations for future research include, therefore, sustainable, practical self-management programmes for black patients with HF living in developing urban areas, where their socio-economic circumstances often remain poor. When combined with other aspects of culturally specific multidisciplinary care, the positive impact of such programmes is likely to be profound.
